# Phthalate and novel plasticizer concentrations in food items from U.S. fast food chains: a preliminary analysis

**DOI:** 10.1038/s41370-021-00392-8

**Published:** 2021-10-27

**Authors:** Lariah Edwards, Nathan L. McCray, Brianna N. VanNoy, Alice Yau, Ruth J. Geller, Gary Adamkiewicz, Ami R. Zota

**Affiliations:** 1 Department of Environmental and Occupational Health, The George Washington University Milken Institute School of Public Health, Washington, DC USA; 2 Department of Analytical and Environmental Chemistry, Southwest Research Institute, San Antonio, TX USA; 3 Department of Epidemiology, Boston University School of Public Health, Boston, MA USA; 4 Department of Environmental Health, Harvard T.H. Chan School of Public Health, Boston, MA USA

**Keywords:** Phthalates, Plasticizers, Dietary exposures, Fast food, Food handling gloves, Health disparities

## Abstract

**Background:**

Fast food consumption is associated with biomarkers of ortho-phthalates exposures. However, the chemical content of fast food is unknown; certain ortho-phthalates (i.e., di-*n*-butyl phthalate (DnBP) and di(2-ethylhexyl) phthalate (DEHP)) have been phased out and replaced with other plasticizers (e.g., dioctyl terephthalate (DEHT)).

**Objective:**

We conducted a preliminary study to examine ortho-phthalate and replacement plasticizer concentrations in foods and food handling gloves from U.S. fast food restaurants.

**Methods:**

We obtained hamburgers, fries, chicken nuggets, chicken burritos, cheese pizza (*n* = 64 food samples) and gloves (*n* = 3) from restaurants and analyzed them for 11 chemicals using gas chromatography mass spectrometry.

**Results:**

We found DEHT at the highest concentrations in both foods (*n* = 19; median = 2510 µg/kg; max = 12,400 µg/kg) and gloves (*n* = 3; range: 28–37% by weight). We detected DnBP and DEHP in 81% and 70% of food samples, respectively. Median DEHT concentrations were significantly higher in burritos than hamburgers (6000 µg/kg vs. 2200 µg/kg; *p* < 0.0001); DEHT was not detected in fries. Cheese pizza had the lowest levels of most chemicals.

**Significance:**

To our knowledge, these are the first measurements of DEHT in food. Our preliminary findings suggest that ortho-phthalates remain ubiquitous and replacement plasticizers may be abundant in fast food meals.

**Impact statement:**

A selection of popular fast food items sampled in this study contain detectable levels of replacement plasticizers and concerning ortho-phthalates. In addition, food handling gloves contain replacement plasticizers, which may be a source of food contamination. These results, if confirmed, may inform individual and regulatory exposure reduction strategies.

## Introduction

Ortho-phthalates (also referred to as phthalates) are a class of multi-functional, high production volume chemicals used widely in commerce [1]. High molecular weight phthalates like di(2-ethylhexyl) phthalate (DEHP) and diisononyl phthalate (DiNP) are commonly used as plasticizers in polyvinyl chloride (PVC) materials such as food packaging and food contact materials [1, 2]. In the past twenty years, the European Union and U.S. have restricted the use of several ortho-phthalates, including DEHP and di-*n*-butyl phthalate (DnBP), in commercial products. As a result, plasticizers such as di(2-ethylhexyl) adipate (DEHA), 1,2-cyclohexane dicarboxylic acid diisononyl ester (DINCH), and dioctyl terephthalate (also known as di(2-ethylhexyl) terephthalate) (DEHT) have emerged as replacement plasticizers in PVC materials [3, 4]. Despite the name, DEHT is structurally distinct from ortho-phthalates.

Human exposure to ortho-phthalates is widespread since they easily migrate out of products. Indeed, biomarkers of phthalates exposures are detected in greater than 98% of the U.S. population [2]. Widespread population exposure is concerning since certain ortho-phthalates are established endocrine disruptors linked to a host of adverse reproductive and metabolic outcomes across the life course [5, 6]. Recently, Project TENDR (Targeting Environmental Neurodevelopmental Risks), which consists of a group of scientists and health professionals with expertize in toxic chemicals and neurotoxicity, concluded that there is substantial evidence linking phthalate exposures to increased risks for children’s learning, attention, and behavioral problems. In addition, Project TENDR recommended that phthalates be eliminated from products that may lead to exposure among vulnerable populations (e.g., pregnant women, children, and communities of color) [7].

Diet is the primary source of exposure for most ortho-phthalates and, potentially, an important source of replacement plasticizer exposures. Reducing dietary exposures to phthalates was one of five critical recommendations in the recent Project TENDR publication [7]. However, current available data do not adequately capture the extent of phthalate and non-phthalate plasticizer contamination across the major components of our diet, particularly for vulnerable populations [8]. Addressing this data gap is essential to protecting the food supply from phthalates. For decades, expenditure data indicate that Americans spend at least 50% of their food budget on restaurant meals [9]. Food items sold by fast food chains, the most common restaurant type in the U.S., are heavily processed, packaged, and handled, therefore, individuals who frequently consume fast food meals are especially vulnerable to plasticizer exposures. Several epidemiologic studies have reported associations between frequent fast food or ultra-processed food consumption and increased exposure to ortho-phthalates using nationally representative data of the U.S. general population [10–13]. Yet, to our knowledge, there are no studies that measure plasticizer concentrations found in fast food items from U.S. restaurants. The concentrations of these chemicals in fast foods represent the culmination of plasticizer exposures across the entire food supply chain.

The lack of data on plasticizers in fast food is particularly concerning from a health equity perspective. Racial residential segregation impacts food landscapes and dietary behavior. For example, predominately Black areas in New York City have higher densities of fast food than predominately White areas, and high-income Black neighborhoods have similar exposure as low-income Black neighborhoods [14]. Relatedly, a Centers for Disease Control and Prevention report showed that fast food consumption was higher among Black people in the U.S. [15]. Our prior work suggests that chemical contamination of food may disproportionally impact marginalized groups since we observed a stronger association between fast food intake and urinary metabolites of DEHP among Non-Hispanic Blacks compared to Non-Hispanic Whites and Hispanics in the U.S. general population [11]. These pathways likely contribute to the pervasive racial/ethnic disparities in chemical exposures [16].

Detection rates of urinary DINCH and DEHT metabolites have steadily increased in the U.S. population in parallel with their increased use as ortho-phthalate substitutes [17, 18]. In a recent report by ChemSec (the International Chemical Secretariat), a Swedish non-profit that advocates for safer alternatives for toxic chemicals in consumer products, DEHT and DINCH were listed as the most common non-phthalate substitutes in the U.S. and Europe, respectively [3]. However, the use of these chemicals in products does not guarantee their safety. The toxicity information for replacement plasticizers is limited to a few animal studies, thus, the human health implications of chronic exposures to replacement plasticizers are poorly understood [19–21]. In lieu of robust animal and human data, in vitro models such as high throughput assay data can be useful for understanding how replacement plasticizers can disrupt cellular pathways and contribute to adverse human health outcomes.

Accordingly, the objective of this preliminary exposure assessment study is to quantify concentrations of eight ortho-phthalates and three replacement plasticizers in food items commonly ordered from popular fast food restaurants. We characterize variability by food type as well as within fast food chains and across sampling phases. We focus on measuring chemical analytes in fast food items, as consumed by the general public. We also quantify chemical concentrations in food handling gloves, a suspected source of plasticizers in fast food [22]. To help advance information on toxicity of the replacement plasticizers, we query and summarize relevant high throughput assay data for each plasticizer downloaded from U.S. EPA Comptox Chemicals Dashboard.

## Materials and methods

### Sampling and data collection

**Fig. 1:**
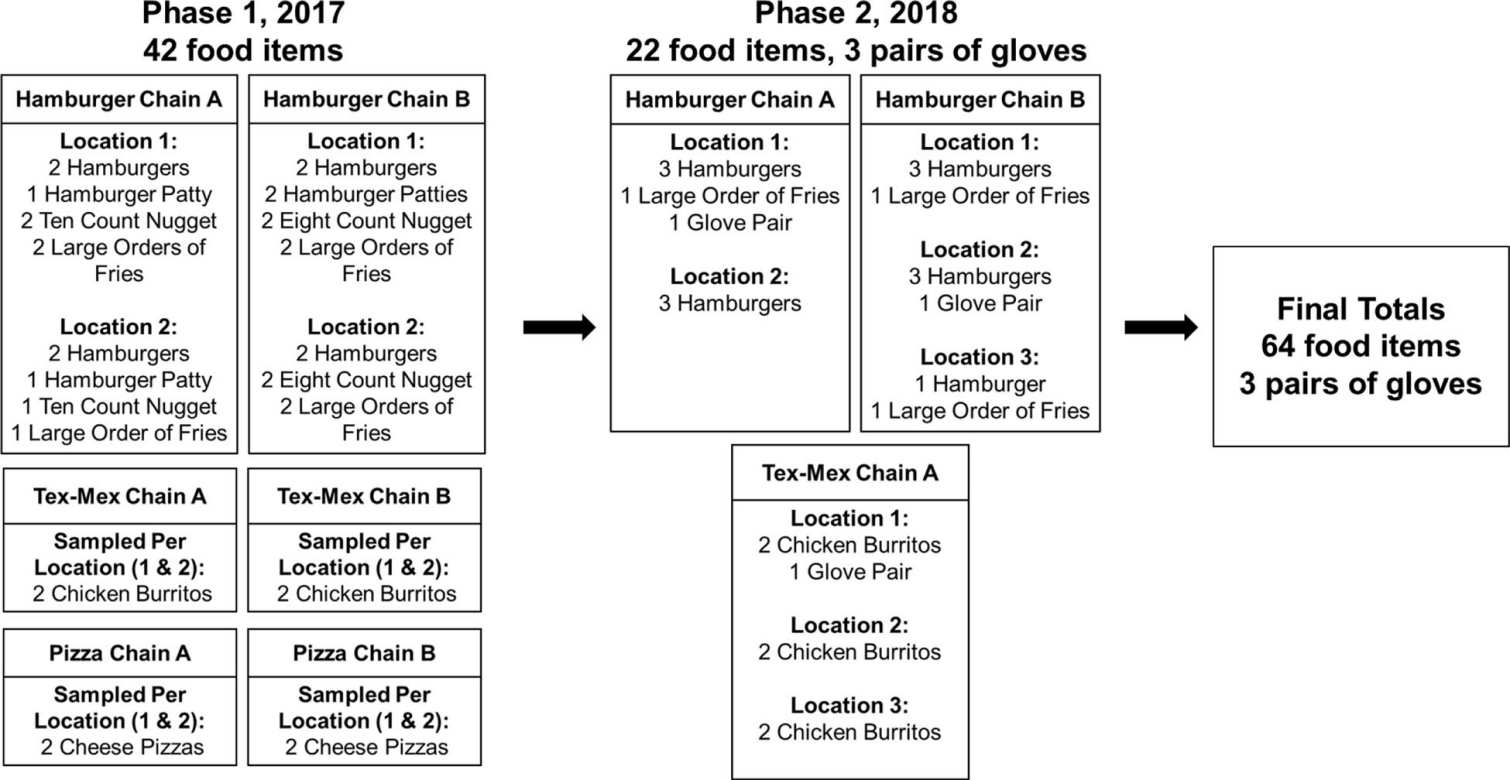
Sampling plan for fast food items and food handling gloves. Fast food chain sampling plan for phase 1 (2017) and phase 2 (2018).

For this preliminary study, we used market share data to select the most popular fast food chains from three food categories: hamburger, pizza, and Tex-Mex [23, 24]. Selected restaurants were ranked in the top five of each category and located in San Antonio, Texas near our analytical laboratory. Restaurant names and rankings are provided in Supplementary Material Table 1. We sampled fast food items from chains in two phases from 2017 to 2018 (Fig. 1). To assess variability in plasticizer concentrations within chain restaurants, we sampled food items from multiple locations per chain (Fig. 1). We used information from the literature, including results from our prior study, to inform our selection of fast food items. In Zota et al. (2016), we found that foods categorized as meats or grains, such as hamburgers, chicken nuggets, and burritos, were associated with higher exposure to DEHP and DiNP [11]. At each restaurant, we sampled the most commonly ordered foods with standard toppings and fillings to represent exposures from popular dietary selections. Information about standard toppings and fillings for each hamburger and chicken burrito is shown in Table 1. In phase 1 (February–March 2017), we sampled hamburgers, chicken nuggets, fries, chicken burritos, and cheese pizzas (*n* = 42) from six restaurants, with two separate locations per chain. Due to limited resources, we sampled a smaller selection of foods in phase 2. Since we observed relatively low chemical concentrations in pizza and chicken nuggets in phase 1, those items were not re-sampled in phase 2. In phase 2 (September 2018), we sampled hamburgers, fries, and chicken burritos (*n* = 22) from three of the same chains, with an additional third location for two of the chains. Food items from each specific location were purchased on the same day. All food items were ordered and packaged separately to avoid cross-contamination and transported to the lab in a cooler in their original packaging. A single polyurethane foam plug served as the field blank at each of the restaurants during each sampling phase. Samples were frozen at −20 °C until analysis. In addition, in phase 2, we collected one pair of gloves from each restaurant (*n* = 3), since gloves are a suspected source of plasticizer contamination. Gloves were collected from the box by an employee, transported in a glass container, and stored at ambient conditions until analysis.

**Table 1:** Frequencies and concentrations (μg/kg) of phthalates and replacement plasticizers detected in foods at selected fast food chains in San Antonio, TX.

Food (*n* = Tot; Ph 1: Ph 2)		Hamburgers^a^ (*n* = 21; 8:13)			French Fries (*n* = 10; 7:3)			Chicken Burritos^a^ (*n* = 14; 8:6)			Cheese Pizza (*n* = 8; 8:0)			Chicken Nuggets (*n* = 7; 7:0)			Hamburger Patties^a^ (*n* = 4; 4:0)			
	MDL	% > MDL	Med	95th Per	% > MDL	Med	95th Per	% > MDL	Med	95th Per	% > MDL	Med	95th Per	% > MDL	Med	95th Per	% > MDL	Med	95th Per	Tot % > MDL
Ortho-Phthalates^b^																				
BBzP	5	29	<MDL	9.9	40	<MDL	26	7	<MDL	5.6	0	<MDL	<MDL	29	<MDL	19	0	<MDL	<MDL	20.3
DnBP	2	100	4.8	7.9	80	4.5	9.1	71	3.5	6	100	4.1	4.8	57	2.4	7.6	25	<MDL	3.3	81.3
DnOP	2	0	<MDL	<MDL	0	<MDL	<MDL	0	<MDL	<MDL	38	<MDL	2.4	14	<MDL	2.3	0	<MDL	<MDL	6.3
DEP	5	38	<MDL	15.8	50	<MDL	25.5	36	<MDL	13	13	<MDL	5.4	29	<MDL	15	100	16.5	25	39.1
DEHP	14	100	36	57.3	70	23.7	84.9	57	23	78.2	0	<MDL	<MDL	100	26	79	50	14	23	70.3
DiBP^c^	1	88	2.7	3.5	57	1.2	1.7	13	<MDL	1.2	50	1.9	3.8	0	<MDL	<MDL	0	<MDL	<MDL	38.1
DiNP^c^	5	0	<MDL	<MDL	0	<MDL	<MDL	100	36*	57	50	5.9*	13	0	<MDL	<MDL	0	<MDL	<MDL	28.6
Replacement Plasticizers																				
DEHA	1	62	6.7*	12.7	30	<MDL	12.7	71	45.7*	170	0	<MDL	<MDL	0	<MDL	<MDL	0	<MDL	<MDL	40.6
DINCH	10^d^	24	<MDL	590	0	<MDL	<MDL	0	<MDL	<MDL	0	<MDL	<MDL	29	<MDL	180	50	16.3	37	14.1
DEHT^e^	50	100	2200*	3200	0	<MDL	<MDL	100	6000*	12,400	ns	ns	ns	ns	ns	ns	ns	ns	ns	86.4

*Tot* total, *MDL* method detection limit, *% > MDL* percent detected above MDL, *Med* median, *Per* percentile, *ns* not sampled, *n/a* not applicable, *<MDL* concentration was below the MDL.

^a^Hamburgers were ordered with cheese, tomatoes, pickles, onions, lettuce and select condiments, and hamburger patties were ordered without any toppings. Burritos from Chain A were ordered with white rice, black beans, cheese, mild salsa, and lettuce, and burritos from Chain B were ordered with refried pinto beans, cheese, onions, sour cream, tomatoes, lettuce, and a tomato-based sauce.

^b^DMP sampled but dropped due to non-detect in all food items.

^c^Sampled in phase 1 only (*n* = 42 total samples).

^d^MDLs varied across phases. Phase 1 = 5 μg/kg; Phase 2 = 10 μg/kg.

^e^Sampled in phase 2 only (*n* = 22 total samples).

*Exact Wilcoxon test for DiNP, DEHA, and DEHT and the Kruskal Wallis test for DnBP, DEHP, and DiBP were used to assess significant differences in individual chemical concentration when comparing across food types. An * indicates that the median phthalate concentration varied by food type (*p* < 0.05).

Concentrations below the MDL were replaced with a value equal to the MDL divided by the square root of two.

### Chemical analysis

Butyl benzyl phthalate (BBzP), di-*n*-butyl phthalate (DnBP), diethyl phthalate (DEP), di(2-ethylhexyl) phthalate (DEHP), dimethyl phthalate (DMP), di-n-octyl phthalate (DnOP) (cat. # ASM-146) and di(2-ethylhexyl) phthalate-3,4,5,6-d4 (DEHP-d4) (cat. # PHTH-D4-011S) solutions were purchased from AccuStandard Inc. (New Haven, CT, USA). Di (2-ethylhexyl) terephthalate (DEHT) (part # S-14065J1) and di (2-ethylhexyl) isophthalate solutions (part # S-11224J1) were purchased from Chem Service Inc. (West Chester, PA, USA). 1,2-cyclohexane dicarboxylic acid diisononyl ester (DINCH) solution was purchased from Matrix Scientific (cat# 095991) (Columbia, SC, USA). Diisononyl phthalate (DiNP) (cat# S-1559, mixture of isomers) and diisobutyl phthalate (DiBP) (cat# S-4150) solutions were purchased from SPEX CertiPrep (Metuchen, NJ, USA). Di(2-ethylhexyl) adipate (DEHA) (cat# 31449) solution was purchased from Restek Corporation (Bellefonte, PA, USA).

We quantified 11 analytes in food samples using gas chromatography mass spectrometry (GC/MS). Samples were analyzed separately by phase. We used retention times to identify phthalates and replacement plasticizers in food samples. Some chemicals were measured in both phases (BBzP, DnBP, DEP, DEHP, DMP, DnOP, DEHA, and DINCH) while other chemicals were only measured in one phase. DiNP and DiBP were only quantified in phase 1 because we generally observed low concentrations of these chemicals in foods sampled in phase 1. Conversely, DEHT was quantified in phase 2 food samples because it was detected on chromatograms during the analysis of phase 1 samples. However, due to limited resources, we were unable to reanalyze phase 1 foods for DEHT.

Food items were weighed and homogenized prior to chemical analysis. The sample preparation process was based on methodology by Tsumura et al. (2001) [25]. Approximately 50 g of the homogenized sample was transferred to a glass jar and 100 mL of acetonitrile was added. The sample was placed in a shaker for 30 min followed by centrifugation. The acetonitrile layer was removed and transferred to another container. A second aliquot of fresh 100 mL acetonitrile was added to the food sample and the extraction process was repeated. The acetonitrile layer was removed and combined with the first aliquot. Next, 7 g of NaCl was added to the acetonitrile and 40 mL of hexanes, previously saturated with acetonitrile, was added to the acetonitrile. After shaking for 30 min, the acetonitrile layer was removed, and concentrated, and the solvent was exchanged to 5 mL hexanes. The hexanes extract was passed through a Florisil and PSA column and the eluent was discarded. The column was rinsed with another 5 ml of hexanes followed by 10 mL of 5% acetone in hexanes. The resulting eluent was collected and concentrated to 5 mL volume prior and used in gas chromatography mass spectrometry analysis. Additional details on the GC/MS analysis are provided in the Supplementary Material. To measure most plasticizers in the food samples, we used a calibration curve range from 0.01 to 2 µg/mL. However, for DINCH and DiNP, we raised the calibration curve range five-fold due to the multi-component nature of the chemicals and the fact that the diluted detections were over the calibration range.

Field blanks from both phases were used to calculate the method detection limit (MDL) of each chemical. If a chemical was detected in the polyurethane foam plug field blank, its MDL was calculated as the field blank mean plus the standard deviation multiplied by three. When chemical concentrations in field blanks varied between phases, we used the phase with the higher concentrations to calculate the chemical-specific MDL. DEHP, BBzP, DEP, DnBP, and DnOP were detected in field blanks, and based on the above criteria, their MDLs were: DEHP: 14 μg/kg, BBzP: 5 μg/kg, DEP: 5 μg/kg, DnBP: 2 μg/kg, and DnOP 2 μg/kg. When there was no detectable concentration of the chemical in the field blank, the chemical’s MDL was equivalent to the analytical limit of quantitation (LOQ). Details on how the chemicals’ LOQs were calculated are presented in the Supplementary Material. The MDLs for the remaining chemicals were: DEHT: 50 μg/kg, DMP: 1 μg/kg, DiBP: 1 μg/kg, DiNP: 5 μg/kg, DEHA: 1 μg/kg, and DINCH: 5 μg/kg. In phase 2, the LOQ for DINCH was raised to 10 μg/kg.

Laboratory method blanks were used throughout the chemical analysis for internal control. These blanks were composed of an acetonitrile and hexane solvent mixture and were included in the extraction process and treated like the samples. BBzP, DnBP, DEHP, and DEP were detected in 50% or more of the laboratory method blanks. Chemical concentrations detected in the blanks are presented in Table S2. To assess precision, we analyzed eight samples in duplicate. The relative percent difference between duplicates was <50%, except for DEP (percent difference range: 6–152%). To assess accuracy, we spiked all food samples (*n* = 64) with DEHP-d4 and measured their recoveries. The percent recovery for DEHP-d4 ranged from 32 to 82%. In addition, we also spiked three samples (hamburger, burrito, and pizza) with all phase 1 analytes. Median spike recoveries across products were within 50–113%, except for DEHP (47%), DINCH (27%), DnOP (49%), and DiNP (24%).

For gloves, ~1 gram of the sample was Soxhlet extracted using 200 mL of dichloromethane (DCM) for >14 h. At the end of extraction, the volume of the DCM extract was adjusted to 200 mL and an aliquot of 1 mL was removed for GC/MS analysis. Additional details on the GC/MS analysis are presented in the Supplementary Material. A laboratory method blank, dichloromethane, was included in the extraction batch and treated the same way as the sample. The calibration curve for DEHT was 1–20 µg/mL; for DINCH, the calibration curve was raised five-fold.

### Statistical analysis

Descriptive statistics of the chemicals were calculated for each food item. We compared median chemical concentrations by food type, food chain, location, and sampling phase for all chemicals detected in at least 50% of food samples. In these analyses, we substituted concentrations below the MDL with a value equal to the MDL divided by the square root of two. We used the Wilcoxon Rank Sum and non-parametric Kruskal Wallis tests to test differences in median chemical concentrations. To facilitate comparison with other studies, we also calculated descriptive statistics using only concentrations detected above the MDL (Supplementary Material, Table S3). All statistical analyses were conducted in SAS 9.4 (SAS Institute, Cary, NC).

### ToxCast analyses

Given the limited toxicity data on replacement plasticizers, we queried high-throughput screening data from the U.S. EPA ToxCast Program for additional toxicity information for DEHT, DEHA, and DINCH [26]. We downloaded high-throughput assay data directly from U.S. EPA ToxCast CompTox Dashboard (https://comptox.epa.gov/dashboard, accessed on 31 March 2021). Downloadable assay results for individual chemicals are classified by a hit-call variable, “active” or “inactive” (meeting or not meeting dose–response criteria), for each assay endpoint.

We limited our focus to assays that measure a nuclear receptor gene target since most of the harmful effects associated with endocrine disrupting chemicals, including ortho-phthalates, can be attributed to a chemical’s interference with signaling pathways mediated by nuclear receptors [27]. Therefore, assays that assessed cell viability, cytotoxicity, proliferation, and non-nuclear receptor related gene targets were excluded. The downloaded data included data quality flags for endpoints. Endpoints with the following quality tags were excluded from our summary: “noisy data”, “borderline active”, “AC50 less than lowest concentration tested”, and “hitcall potentially confounded by curve overfitting”. We also excluded two Attagene assays measuring activity for the aryl hydrocarbon receptor and the pregnane X receptor response element (ATG_Ahr_CIS_dn, ATG_PXRE_CIS_dn) because the assay information file stated that these assays were “not developed or optimized to detect loss of signal. Use data with caution” (downloaded from https://www.epa.gov/chemical-research/exploring-toxcast-data-downloadable-data, accessed on 31 March 2021).

## Results and discussion

### Results

We detected ortho-phthalates or replacement plasticizers in all food samples (*n* = 64) (Table 1). DnBP was the most frequently detected ortho-phthalate in foods at 81%, followed by DEHP at 70%. Both were detected in all hamburgers sampled in both phases (Fig. S1). BBzP, DnOP, and DEP were detected in 20%, 6%, and 39% of foods, respectively. DMP was the only chemical not detected in any of the food samples. DiBP and DiNP, phase 1 analytes only, were detected in 38% and 29% of food samples (*n* = 42). Replacement plasticizers, DEHA and DINCH, were detected in 41% and 14% of foods, respectively. DEHT, a phase 2 analyte only, was detected in 86% of all foods (*n* = 22) (Table 1).

Median concentrations of chemicals detected in food samples ranged from below the MDL to 6000 µg/kg (Table 1). The highest observed median chemical concentration in foods was DEHT in burritos (*n* = 6; median = 6000 μg/kg; 95th percentile = 12,400 μg/kg). We also observed elevated concentrations of DINCH in hamburgers (*n* = 5; median = 7.1 μg/kg; 95th percentile = 590 μg/kg). Among the ortho-phthalates, DEHP and DiNP had the highest median concentrations at 36.0 μg/kg in hamburgers and burritos, respectively (Table 1). Generally, foods containing meat had higher chemical concentrations than non-meat foods, particularly for the replacement plasticizers. Relative to other food types, cheese pizza had the lowest concentrations of nearly all chemicals.

Concentrations of DnBP and DEHP were statistically similar across food items. Among ortho-phthalates, only DiNP varied significantly by food type (36 μg/kg burritos vs. 5.9 μg/kg pizza; *p* = 0.002). In contrast, DEHA and DEHT varied by food type (Table 1). For both chemicals, concentrations were significantly higher in burritos compared to hamburgers [(DEHA: 45.7 μg/kg burritos vs. 6.7 μg/kg hamburgers; *p* = 0.006) (DEHT: 6000 μg/kg burritos vs. 2200 μg/kg hamburgers; *p* = <0.0001)].

Concentrations of ortho-phthalates, but not replacement plasticizers, varied slightly across chains (Table S4) and between phases (Table S5). Median concentrations of DnBP and DEHP in foods were significantly different across hamburger chains (*p* = 0.0002 and *p* = 0.005, respectively), and median concentrations of DiNP varied across Tex-Mex chains (*p* = 0.03) (Table S4). In addition, median concentrations of DnBP and DEHP in hamburgers, fries, and burritos combined were significantly higher in phase 2 compared to phase 1 (DnBP: *p* = 0.005; DEHP: *p* = 0.0001) (Table S5).

**Fig. 2:**
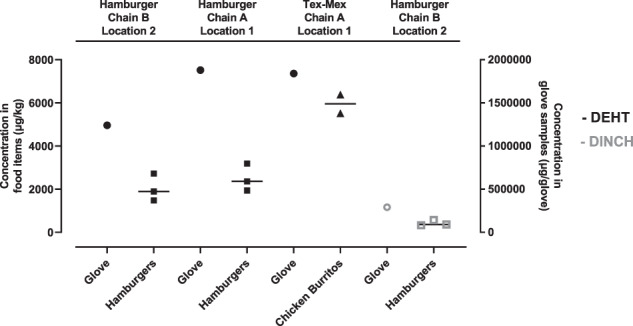
DEHT and DINCH were detected in hamburgers, burritos, and gloves. Fries were collected from Hamburger Chain A only. Comparison of plasticizers DEHT (represented by black symbols) and DINCH (represented by gray symbols) found in gloves and food items sampled from selected fast food restaurants in San Antonio, TX. Gloves and food items were sampled from the same restaurant location and at the same time. For gloves, one pair was collected from each restaurant and individual data from one sample are plotted (µg/glove). For the food items, individual data are plotted with the median indicated by a line (µg/kg).

DEHT and DINCH were detected in both glove pairs and food samples collected from the same restaurant chain and location (Fig. 2). DEHT was detected in all three pairs of food handling gloves collected from Hamburger Chains A and B and Tex-Mex Chain A, with concentrations ranging from 12,40,000–18,80,000 μg/glove or 28–37% by weight (% wt) (Fig. 2). DEHT was detected in hamburgers from Chain A and Chain B, but not in fries collected from Chain A, and in burritos from the Tex-Mex Chain A. DINCH was only detected in hamburgers and gloves from Chain B (glove = 290,000 μg/glove or 7% wt; median hamburgers = 364.1 μg/kg).

DEHT, DINCH, and DEHA were active in four, three, and five ToxCast assays specific to nuclear receptor target genes, respectively. After excluding assays with certain data quality flags, the number of active assays decreased to two for DEHA and one each for DINCH and DEHT. Based on ToxCast data, both DEHT and DEHA were active in assays related to agonist activity for the retinoid x receptor beta (RXBβ) (Table 2). In addition, DEHA was active in an assay related to the antagonist activity for the estrogen receptor alpha (ERα). DINCH activated an assay measuring agonist activity for the pregnane X receptor (PXR).

**Table 2:** Summary of active ToxCast assay data targeting nuclear receptors for replacement plasticizers, DEHT, DINCH, and DEHT.

Gene name	ToxCast assay name^a^	DEHT	DINCH	DEHA
Retinoid X receptor-beta (RXRβ)	ATG_RXRb_TRANS_up	**×**		**×**
Pregnane X receptor (PXR)	ATG_PXRE_CIS_up		**×**	
Estrogen receptor (ERα)	TOX21_ERa_BLAAntagonist_ratio			**×**

^a^Assays shown here represent all active assays for each chemical, excluding non-nuclear receptor target gene assays and assays with certain data quality flags.

### Discussion

In this preliminary exposure assessment study, we report detectable levels of plasticizers in all foods sampled from several major U.S. fast food chains. DnBP and DEHP were the most frequently detected ortho-phthalates. DEHT was the most frequently detected replacement plasticizer and was detected at much higher concentrations than other chemicals. We generally observed higher chemical concentrations in foods containing meat relative to other foods, such as cheese pizza. In addition, we report concentrations of DEHT and DINCH in both foods and gloves sampled from the same restaurant; to our knowledge these are the first reportable data of DEHT in fast food items. Our study adds to prior research by focusing on fast food meals, an important component of cumulative dietary phthalate exposures, that have been highly processed, packaged, and handled. To date, a majority of the studies connecting fast food consumption to ortho-phthalate exposure have relied on biomonitoring data. Our study supports the results from these biomonitoring studies and provides exposure data that can be used in phthalate risk assessments. Since multiple phthalates contribute to common adverse health effects and humans are simultaneously exposed to multiple phthalates, the National Academies recommended that regulatory agencies consider a cumulative risk assessment approach for ortho-phthalates [1]. To this end, accurate data from all potential exposure sources are critical. Furthermore, until the U.S. takes regulatory action to address phthalate contamination in foods, these findings could push restaurants to voluntarily adopt policies to eliminate harmful chemicals in their foods.

While several prior studies have measured concentrations of ortho-phthalates and plasticizers in foods, many are not directly comparable to our study due to differences in the food items sampled or the sources of foods (i.e., grocery store or market). One prior study measured DEHT in 14 milk and dairy supermarket products using analytical methods similar to our study and reported that all DEHT concentrations were below the LOQ of 0.05 µg/g [28]. One potentially relevant study is by Cao et al. (2015) where they quantified the concentrations of DiBP, DnBP, DEHP, and DEHA, in pizza, fries, chicken nuggets, and hamburgers as part of the Canadian 2013 Total Diet Study and used analytical methods similar to our study [29]. However, there are important differences in study design since the authors focused on food items from grocery stores rather than a restaurant. We generally report lower mean concentrations of the ortho-phthalates in our sampled foods than Cao et al. (2015) except for DEHP concentrations in hamburgers, which were similar between the two studies (our study: 40.3 µg/kg vs. Cao et al. 2015: 43.0 µg/kg) [29]. Our results are similar to data presented in a recent review article, which summarized concentrations of ortho-phthalates in different foods using data published from 2001 to 2019 [30]. The authors reported high concentrations of DnBP and DEHP in beef, cheese, and oils, butters, and fats; DEHP was also detected in high concentrations in chicken [30]. These results align with our findings of generally higher chemical concentrations in meals containing meat.

To our knowledge, only one other study has reported DEHT and DINCH concentrations in food handling gloves commonly used at U.S. based fast food restaurants. In a white paper by the Ecology Center, the authors collected 101 vinyl (non-medical) gloves from distributors that supply restaurants, including several restaurants sampled in our study [31]. Overall, the authors reported both ortho-phthalates and non-phthalate plasticizers in gloves. The authors used GC/MS to quantify DEHT and DINCH in three glove samples that did not contain ortho-phthalates and reported that their gloves contained an average of 32.3% wt of DEHT (*n* = 3) and 2.6% wt of DINCH (*n* = 1) [31]. In our study, we report a similar average DEHT concentration from gloves collected from three fast food chains (our study: *n* = 3; 33% wt) and a slightly higher DINCH concentration (*n* = 1; 7% wt).

In addition, our findings of DEHT and DINCH in foods collected from the same restaurants as our gloves support the assertion that food handling contact materials may be one source of plasticizer exposure. A study by Tsumura et al. (2001) reported higher levels of DEHP in foodstuffs after they were handled and packaged with PVC gloves containing DEHP [22]. We measured high concentrations of DEHT in hamburgers and burritos and DINCH in hamburgers, but neither was detected in fries. Hamburgers and burritos require more assembly than fries, thus the increased handling of hamburgers and burritos by gloved workers may explain their higher concentrations. Alternatively, this finding could reflect the generally higher concentrations of chemicals in foods with meat. Overall, our detection of plasticizers in foods from U.S. chains is consistent with recent biomonitoring studies that have detected urinary metabolites of DEHA, DEHT, and DINCH in various study populations and report that DEHT exposure is increasing in the general population over time [4, 17, 18, 32].

The widespread detection of DEHP and DnBP in our sample of popular fast foods, but not in gloves, may suggest that contamination of foods occurs at other parts of the food supply chain. Recently, a study conducted by U.S. Food and Drug Administration (FDA) scientists reported detectable concentrations of six ortho-phthalates (DnBP, DEHP, BBzP, DINP, DnOP, and diisodecyl phthalate [DIDP]) in paper-based fast food packaging collected from restaurants located in Washington, D.C. [33]. Most concentrations were below the MDL or upper confidence limit. However, DEHP, DnBP, BBzP, and DiNP were detected in food packaging samples from fries, hamburgers, pizza, and chicken; similar foods were also included in our study. Another study by the same group analyzed PVC food contact and food processing materials for ortho- and non-phthalate plasticizers and reported detectable concentrations of DEHP in tubing and DiNP in conveyor belts [34]. By focusing exclusively on specific food contact materials, the authors are characterizing chemical exposure at particular parts of the food supply chain, and simultaneously, neglecting the combined contribution of all potential sources of plasticizer exposure. In contrast, the chemical concentrations we quantified in our study of fast food items likely represent contamination from multiple sources, including those analyzed in the FDA studies, as well as other sources not considered in the FDA studies.

Widespread phthalate exposure, including potential contamination of the food supply, is concerning for human health. Exposure to ortho-phthalates such as DEHP and DnBP is linked to adverse health effects including neurodevelopmental, metabolic, and reproductive disorders [5, 7, 35, 36]. For example, DEHP is a well-known male reproductive toxicant and induces cryptorchidism and changes in testicular testosterone and Leydig cell homeostasis [37]. This body of evidence has prompted a consumer push for phthalate-free products and regulatory actions to limit the use of ortho-phthalates in commercial products. However, unlike the ortho-phthalates, there is limited toxicity and health evidence for the replacement plasticizers, and research suggests that these replacements are increasing in use before their health effects are well characterized. In rats, DEHA was a mild to moderate developmental toxicant, however, anti-androgenic or reproductive effects were not observed [21, 38–40]. Similarly, there have been no reports of reproductive effects or carcinogenicity in rats exposed to DEHT, although a majority of these findings come from industry-funded publications [20, 41–43] and the results should be interpreted with caution [44]. The reproductive toxicity of DINCH is unclear. Two experimental studies reported effects on the testes and Leydig cells following DINCH exposure, while another study reported no alterations to male reproductive function [19, 45, 46]. In addition, a few epidemiologic studies have linked urinary metabolites of DINCH and DEHT to uterine fibroids and oxidative stress and inflammation, however, the results to date lack consensus [47–49].

The ToxCast data for the replacement plasticizers DEHT, DINCH, and DEHA suggest that these chemicals interact with one or more of the following nuclear receptor signaling pathways: RXRβ, PXR, and ERα. These nuclear receptors play significant roles in human health, such that abnormal activity could perturb normal physiological functioning and lead to adverse health effects. The retinoid X receptors (RXR) serve as heterodimerzation partners to one-third of the receptors in the nuclear receptor superfamily. Therefore, activation of RXR by ligands plays an important regulatory role in multiple nuclear receptor signaling pathways and inappropriate activation by environmental chemicals can lead to multiple adverse human health outcomes [27]. The pregnane X receptor (PXR), also known as the steroid and xenobiotic X receptor, is involved in metabolism and detoxification of drugs and exogenous chemicals. In addition, PXR activation has been linked to health outcomes such as colon and hepatic cancers and chemoresistance [27, 50]. The estrogen receptors (ER) have been widely studied in relation to human health diseases, in part because they are involved in the regulation of many complex physiological processes. As a result, disruptions to the ER signaling pathway contribute to cardiovascular and metabolic diseases and certain cancers [27]. This evidence helps illustrate how the replacement plasticizers, potentially via these specific nuclear receptors, could lead to adverse health outcomes. Importantly, this evidence also highlights the need for more rigorous and concrete data. In fact, according to GreenScreen for Safer Chemicals ratings, an assessment method for scientifically judging the quality of chemical substitutes to avoid regrettable substitutions from the NGO Clean Production Action, DEHT and DINCH are good substitutes for ortho-phthalates, but the organization advocates for finding safer alternatives [3].

Our findings must be considered in light of the strengths and limitations of this study. This is the first study to quantify concentrations of ortho-phthalates and replacement plasticizers in food and gloves from U.S. fast food chains and the first to detect DEHT in foods. However, since this is a preliminary study, there are some important limitations. We had small sample sizes, particularly for the DEHT analysis, which limited our ability to conduct some statistical analyses. We only sampled the most popular foods from each restaurant and all the restaurants were located in one city, so our findings may not be generalizable to all meals served at all fast food restaurants. In this preliminary study, the quantification of chemicals in food handling gloves lacked precision. Due to limitations in our extraction methods, we were only able to detect chemicals present in gloves at high concentrations. In addition, chemical concentrations in the gloves may have decreased since the time the glove was manufactured, so we are likely underestimating the concentrations of DEHT and DINCH in our glove samples. We detected some variability in our concentrations by sampling phase, which may be a result of seasonality since phase 1 and phase 2 samples were collected in different seasons (i.e., spring vs. fall). However, the sample concentrations were not consistently different between phases. Although useful for screening purposes, the ToxCast assay data is generated using an in vitro system which cannot fully capture the bioactivity of a chemical metabolized in a whole organism. As a result, we cannot make definitive conclusions about the toxicity of DEHA, DINCH, and DEHT based solely on the ToxCast assay results.

## Conclusions

Our preliminary findings suggest that ortho-phthalates and replacement plasticizers, like DEHT, are abundant in prepared meals available at popular fast food restaurants. These data support prior observations that consumption of highly processed and prepared foods contribute to human exposure of legacy ortho-phthalates [10–13]. Many of these chemicals have been associated with adverse health outcomes or based on in vitro data, have the potential to be harmful to human health. These results have implications for health equity since Black people in the U.S. report greater fast food consumption than other racial/ethnic groups and also face higher exposures to environmental chemicals from other sources [11, 15, 16]. Furthermore, these results, if confirmed, can inform individual, market-based, and regulatory exposure reduction strategies and support environmental public health prevention.

## Supplementary information

Supplementary Information
