# Supplementary material for: Phthalate and novel plasticizer concentrations in food items from U.S. fast food chains: a preliminary analysis
Source: J Expo Sci Environ Epidemiol. 2021 Oct 27;32(3):366–73. doi: 10.1038/s41370-021-00392-8 (PMC9119856; doi:10.1038/s41370-021-00392-8)
Supplement: Supplementary file 1 — Supplementary Information [file 41370_2021_392_MOESM1_ESM.docx]

**Supplemental Material**

**Phthalate and Novel Plasticizer Concentrations in Food Items from U.S. Fast Food Chains: A Preliminary Analysis**

Lariah Edwards, Ph.D.^1^, Nathan L. McCray, M.P.H^1^, Brianna N. VanNoy, M.P.H.^1^, Alice Yau, Ph.D.^2^, Ruth J. Geller, M.H.S.^1,3^, Gary Adamkiewicz, Ph.D., M.P.H.^4^, Ami R. Zota, Sc.D., M.S.^1^

^1^ Department of Environmental and Occupational Health, The George Washington University Milken Institute School of Public Health, Washington DC, USA

^2^ Department of Analytical and Environmental Chemistry, Southwest Research Institute, San Antonio, TX, USA

^3^ Department of Epidemiology, Boston University School of Public Health, Boston, MA USA

^4^ Department of Environmental Health, Harvard T.H. Chan School of Public Health, Boston, MA, USA

**Address correspondence to:** Ami R. Zota, Sc.D., M.S.**,** Department of Environmental and Occupational Health**,** Milken Institute School of Public Health**,** The George Washington University**,** 950 New Hampshire Avenue NW**,** Washington, DC 20052**,** Phone: 202-994-9289

Email: azota@gwu.edu

Table of Contents

**Supplemental Methods: additional details on GC/MS analysis**

**Figure S1.** Comparison of ortho-phthalates DnBP and DEHP detected in hamburgers from selected fast food restaurants in San Antonio, TX. Plots show individual data plotted with the median indicated by a line (µg/kg) by fast food chain and phase for: a) DEHP and B) DnBP.

**Table S1.** Top 5 restaurants within each category, hamburgers, pizza, and Tex-Mex. Restaurants listed in bold are included in this study.

**Table S2.** Comparison of average concentrations (μg/kg) of ortho-phthalates and replacement plasticizers detected in laboratory method blanks and field blanks.

**Table S3.** Frequencies and concentrations (μg/kg) of ortho-phthalates and replacement plasticizers detected in foods from selected fast food chains in San Antonio, TX.

**Table S4.** Comparison by restaurant chain of median chemical concentrations (μg/kg) of ortho-phthalates and replacement plasticizers detected in foods from selected fast food chains in San Antonio, TX.

**Table S5.** Comparison by phase of median concentrations (μg/kg) of ortho-phthalates and replacement plasticizers in all food types (hamburgers, fries, and chicken burritos) sampled in both phase 1 and 2

**Supplemental Methods**

Gas chromatography/mass spectrometry (GC/MS) analysis of the fast food samples was conducted using Agilent 6890 GC with 5973 single quadrupole mass spectrometer. The mass spectrometer was set in selected ion monitoring (SIM) mode, and a Agilent DB-5 MS (30 m; 0.25 mm id; 0.25 µm film) capillary column used. The oven program started at 60 °C, the temperature was held there for 1 minute, and gradually increased by 10°C per minute to a final temperature of 310 °C. The injector temperature was set at 270 °C. Two to three ions were monitored for each of the analytes. The table below contains the ions used for quantitation and confirmation.

| **Analyte** | **Quantifier ion** | **Confirmation ions** |
| --- | --- | --- |
| Dimethyl phthalate (DMP) | 163 | 164, 194 |
| Diethyl phthalate (DEP) | 149 | 177, 222 |
| Di-n-butyl phthalate (DnBP) | 149 | 205, 223 |
| Benzylbutyl phthalate (BBzP) | 149 | 91, 206 |
| Diethylhexyl phthalate (DEHP) | 149 | 167, 279 |
| Di-n-octyl phthalate (DnOP) | 149 | 279 |
| Diisononyl phthalate (DiNP) | 149 | 167, 293 |
| Diisobutyl phthalate (DiBP) | 149 | 205, 223 |
| 1,2-cyclohexane dicarboxylic acid diisononyl ester (DINCH) | 155 | 127, 281 |
| Diethylhexyl adipate (DEHA) | 129 | 147, 241 |
| Di (2-ethylhexyl) terephthalate (DEHT) | 149 | 167, 261, 279 |

The reporting limit for each phthalate was calculated using the following equation:

Analytical LOQ= amount of food used for extraction × lowest concentration of phthalate × final volume of sample extract for analysis

50 g of each food item was used for extraction. For single component phthalates, the lowest level was 0.01 µg/mL. The concentrations were raised five-fold to 5 µg/kg for multi component phthalates (DINCH and DiNP). The final volume of the extract used in the GC/MS analyses was 5 mL.

Calibration curve ranged from 10 – 500 ng/mL for single component analytes (BBzP, DMP, DnBP, DnOP, DiBP, DEP, DEHP, DEHA). We raised the curve 5-10 fold higher for compounds such as DINCH and DiNP which contained multiple isomers. We used linear regression models to quantitate the target analytes in the sample extracts. Analytes with concentrations that exceeded the calibration curve were diluted for reanalysis.

*Food Handling Gloves*

The analysis of food handling gloves was conducted in full scan mode and the mass spectrometer was set to scan from m/z 45 – 525. The calibration range was increased and the level ranged from 1 – 50 µg/mL.


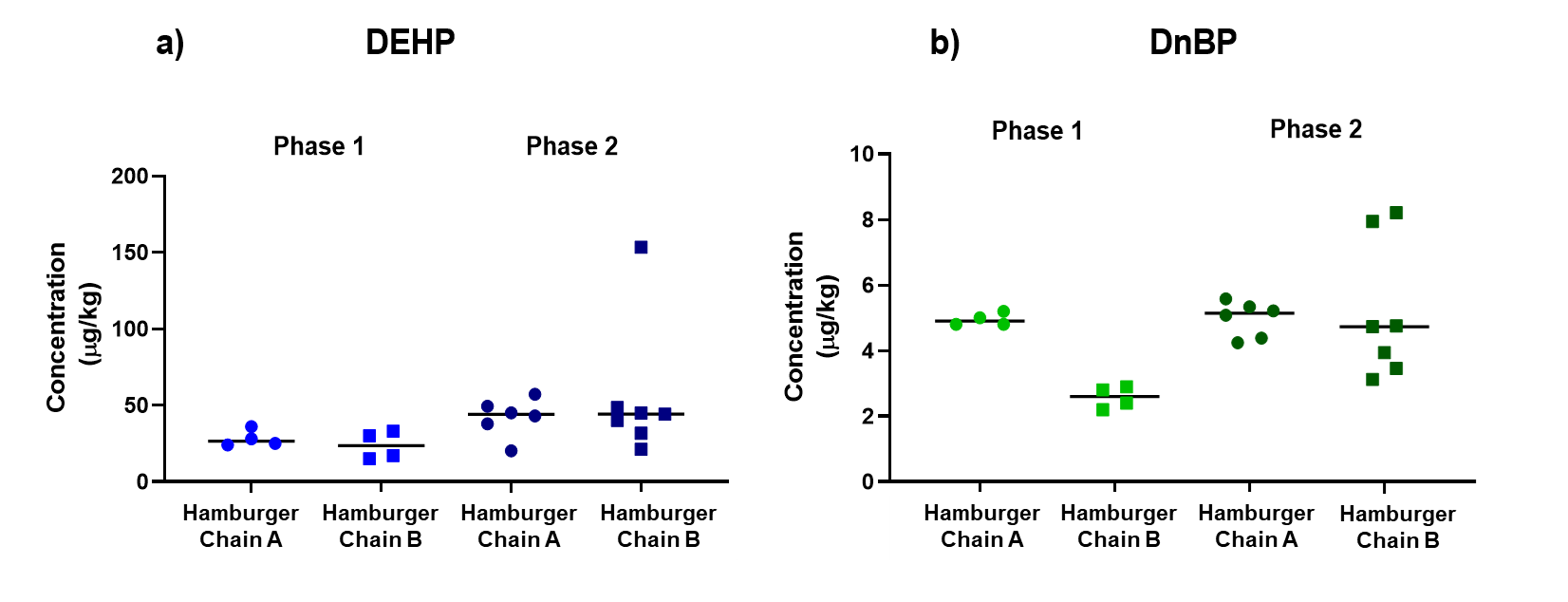


**Figure S1.** Comparison of ortho-phthalates DnBP and DEHP detected in hamburgers from selected fast food chains in San Antonio, TX. Plots show individual data plotted with the median indicated by a line (µg/kg) by restaurant chain and phase for: a) DEHP and B) DnBP.

**Table S1.** Top 5 restaurants within each category, hamburgers, pizza, and Tex-Mex. Restaurants listed in bold are included in this study.

| **Top U.S. Burger-based Fast Food Chains^a^** | **Top U.S. Pizza Chains^b^** | **Top U.S. Mexican Chains^a^** |
| --- | --- | --- |
| 1. **McDonald’s^c^** | 1. **Pizza Hut** | 1. **Taco Bell** |
| 2. **Burger King** | 2. **Domino’s** | 2. **Chipotle** |
| 3. Wendy’s | 3. Little Caesers | 3. Qdoba |
| 4. Sonic Drive-in | 4. Papa John’s | 4. Del Taco |
| 5. Carl’s Jr./Hardee’s | 5. Papa Murphy’s | 5. Moe’s Southwest Grill |

^a^QSR Magazine, Burger Segment Breakdown and Mexican Segment Breakdown

^b^Market Realist

^c^Restaurants listed in bold were included in the study

**Table S2.** Comparison of average concentrations (μg/kg) of ortho-phthalates and replacement plasticizers detected in laboratory method blanks and field blanks.

| **Chemical** | **Laboratory Method Blank^a^** | **Phase 1 Field Blanks** | **Phase 2 Field Blanks** |
| --- | --- | --- | --- |
| BBzP | 2.6 | 2.7 | -- |
| DEHP | 4.1 | 3.8 | 0.4 |
| DEP | 6.1 | 2.5 | 0.4 |
| DnBP | 1.3 | 1.4 | 0.3 |
| DnOP | 1.4 | 1.5 | -- |
| DEHA | 3.2 | -- | -- |

^a^Concentrations of chemicals detected in laboratory method blanks averaged across both phase 1 and phase 2

**Table S3**. Frequencies and concentrations (μg/kg) of ortho-phthalates and replacement plasticizers detected in foods from selected fast food chains in San Antonio, TX.

|  |  | **Ortho-Phthalates^a^ (n=8)** | | | | | | | **Replacement Plasticizers (n=3)** | | |
| --- | --- | --- | --- | --- | --- | --- | --- | --- | --- | --- | --- |
|  |  | **Sampled in**  **Phases 1 and 2** | | | | | **Sampled in**  **Phase 1** | | **Sampled in**  **Phases 1 and 2** | | **Sampled in Phase 2** |
| **Foods (n=Tot; Ph 1; Ph2)** | **Statistic** | **BBzP** | **DnBP** | **DnOP** | **DEP** | **DEHP** | **DiBP**^b^ | **DiNP**^b^ | **DEHA** | **DINCH** | **DEHT**^c^ |
| **Hamburgers^e^ (n=21; 8:13)** | *MDL* | *5* | *2* | *2* | *5* | *14* | *1* | *5* | *1* | *10^d^* | *50* |
|  | % >MDL | 29 | 100 | - | 38 | 100 | 88 | - | 62 | 24 | 100 |
|  | 5^th^ Per | 5.5 | 2.4 | - | 6.0 | 17.0 | 1.9 | - | 6.1 | 331.2 | 600 |
|  | 25^th^ Per | 5.6 | 3.5 | - | 8.5 | 25.0 | 2.2 | - | 6.9 | 364.2 | 1,940 |
|  | Median | 7.2 | 4.8 | - | 10.6 | 36.0 | 3 | - | 8.8 | 569.2 | 2,200 |
|  | 75^th^ Per | 9.9 | 5.2 | - | 15.0 | 45.0 | 3.4 | - | 9.5 | 590 | 2,510 |
|  | 95^th^ Per | 10.6 | 7.9 | - | 49.7 | 57.3 | 3.5 | - | 13.2 | 670 | 3,200 |
| **Fries**  **(n=10; 7:3)** | % >MDL | 40 | 80 | - | 50 | 70 | 57 | - | 30 | - | - |
|  | 5^th^ Per | 17.0 | 2.2 | - | 6.4 | 15.0 | 1.2 | - | 5.9 | - | - |
|  | 25^th^ Per | 17.5 | 4.1 | - | 7.5 | 21.9 | 1.3 | - | 5.9 | - | - |
|  | Median | 20.8 | 4.9 | - | 7.6 | 44 | 1.4 | - | 6.1 | - | - |
|  | 75^th^ Per | 24.8 | 6.5 | - | 11 | 82.0 | 1.6 | - | 5.9 | - | - |
|  | 95^th^ Per | 26 | 9.1 | - | 25.5 | 84.9 | 1.7 | - | 12.7 | - | - |
| **Chicken Burritos^e^ (n=14; 8:6)** | % >MDL | 7 | 71 | - | 36 | 57 | 13 | 100 | 71 | - | 100 |
|  | 5^th^ Per | 5.6 | 3.0 | - | 5.7 | 22.0 | 1.2 | 10.0 | 31.5 | - | 5,240 |
|  | 25^th^ Per | 5.6 | 3.4 | - | 6.3 | 30.9 | 1.2 | 11.5 | 43.2 | - | 5,370 |
|  | Median | 5.6 | 5.3 | - | 8.5 | 58.6 | 1.2 | 36.0 | 63.4 | - | 6,000 |
|  | 75^th^ Per | 5.6 | 5.3 | - | 11 | 72.5 | 1.2 | 53.0 | 31.5 | - | 9,680 |
|  | 95^th^ Per | 5.6 | 6 | - | 13 | 78.2 | 1.2 | 57.0 | 170 | - | 12,400 |
| **Cheese Pizza**  **(n=8; 8:0)** | % >MDL | - | 100 | 38 | 13 | - | 50 | 50 | - | - | ns |
|  | 5^th^ Per | - | 3.3 | 2.2 | 5.4 | - | 3.1 | 8.3 | - | - | ns |
|  | 25^th^ Per | - | 3.6 | 2.2 | 5.4 | - | 3.4 | 8.9 | - | - | ns |
|  | Median | - | 4.1 | 2.4 | 5.4 | - | 3.7 | 10.7 | - | - | ns |
|  | 75^th^ Per | - | 4.3 | 2.4 | 5.4 | - | 3.8 | 12.5 | - | - | ns |
|  | 95^th^ Per | - | 4.8 | 2.4 | 5.4 | - | 3.8 | 13 | - | - | ns |
| **Chicken Nuggets (n=7; 7: 0)** | % >MDL | 29 | 57 | 14 | 29 | 100 | - | - | - | 29 | ns |
|  | 5^th^ Per | 19 | 2.4 | 2.3 | 9.0 | 19.0 | - | - | - | 170 | ns |
|  | 25^th^ Per | 19 | 3.4 | 2.3 | 9 | 20.0 | - | - | - | 170 | ns |
|  | Median | 19 | 5.8 | 2.3 | 12.0 | 26.0 | - | - | - | 175 | ns |
|  | 75^th^ Per | 19 | 7.4 | 2.3 | 15.0 | 55.0 | - | - | - | 180 | ns |
|  | 95^th^ Per | 19 | 7.6 | 2.3 | 15 | 79.0 | - | - | - | 180 | ns |
| **Hamburger Patties**  **(n=4; 4:0)** | % >MDL | - | 25 | - | 100 | 50 | - | - | - | 50 | ns |
|  | 5^th^ Per | *-* | 3.3 | *-* | 16.5 | 19 | - | - | - | 29 | ns |
|  | 25^th^ Per | *-* | 3.3 | *-* | 12.5 | 23 | - | - | - | 29 | ns |
|  | Median | *-* | 3.3 | *-* | 16.5 | 19 | - | - | - | 33 | ns |
|  | 75^th^ Per | *-* | 3.3 | *-* | 22 | 23 | - | - | - | 37 | ns |
|  | 95^th^ Per | *-* | 3.3 | *-* | 25 | 23 | - | - | - | 37 | ns |

Tot= total; MDL=method detection limit; % > MDL= percent detected above MDL; Med= median; Per=percentile; ns=not sampled; Missing values indicate that the chemical was not detected above its MDL.

^a^DMP sampled but dropped due to non-detect in all food items.

^b^Sampled in phase 1 only (n=42 total samples).

^c^Sampled in phase 2 only (n=22 total samples).

^d^MDLs varied across phases. Phase 1=5 μg/kg; Phase 2=10 μg/kg.

^e^Hamburgers were ordered with cheese, tomatoes, pickles, onions, lettuce, and select condiments, and hamburger patties were ordered without any toppings. Burritos from Chain A were ordered with white rice, black beans, cheese, mild salsa, and lettuce, and burritos from Chain B were ordered with refried pinto beans, cheese, onions, sour cream, tomatoes, lettuce, and tomato-based sauce.

| **Table S4.**  Comparison by restaurant chain of median chemical concentrations (μg/kg) of ortho-phthalates and replacement plasticizers detected in foods from selected fast food chains in San Antonio, TX. | | | | | | | | | | | | | | | |
| --- | --- | --- | --- | --- | --- | --- | --- | --- | --- | --- | --- | --- | --- | --- | --- |
|  | **Hamburger Chains** | | | | | **Tex-Mex Chains** | | | | | **Pizza Chains** | | | | |
|  | **Chain A (n=19)** | | **Chain B**  **(n=23)** | |  | **Chain A (n=10)** | | **Chain B**  **(n=4)** | |  | **Chain A**  **(n=4)** | | **Chain B**  **(n=4)** | |  |
|  | %  >  MDL | Med^a^ | %  >  MDL | Med | *p*-value^b^ | %  >  MDL | Med | %  >  MDL | Med | *p*-value | %  >  MDL | Med | %  >  MDL | Med | *p*-  value |
| **Ortho-Phthalates** | | | | | | | | | | | | | | | |
| **BBzP** | 42 | <MDL | <1 | <MDL | -- | 10 | <MDL | 0 | <MDL | -- | 0 | <MDL | 0 | <MDL | -- |
| **DnBP** | 95 | 5.0 | 70 | 2.8 | 0.0002 | 60 | 4.5 | 100 | 3.4 | 0.60 | 100 | 3.9 | 100 | 4.3 | 0.29 |
| **DnOP** | <1 | <MDL | <1 | <MDL | -- | 0 | <MDL | 0 | <MDL | -- | 75 | 2.3 | 0 | <MDL | -- |
| **DEP** | 53 | 7.5 | 39 | <MDL | -- | 40 | <MDL | 25 | <MDL | -- | 0 | <MDL | 0 | <MDL | -- |
| **DEHP** | 95 | 44.0 | 83 | 23.0 | 0.005 | 60 | 48.2 | 50 | 15.9 | 0.26 | 0 | <MDL | 0 | <MDL | -- |
| **DiBP^c^** | 33 | <MDL | 50 | <MDL | -- | 0 | <MDL | 25 | <MDL | -- | 0 | <MDL | 100 | 3.7 | -- |
| **DiNP^c^** | 0 | <MDL | 0 | <MDL | -- | 100 | 11.5 | 100 | 53.0 | 0.03 | 0 | <MDL | 100 | 10.7 | -- |
| **Replacement Plasticizers** | | | | | | | | | | | | | | | |
| **DEHA** | 37 | <MDL | 39 | <MDL | -- | 100 | 63.4 | 0 | <MDL | -- | 0 | <MDL | 0 | <MDL | -- |
| **DINCH** | <1 | <MDL | 39 | <MDL | -- | 0 | <MDL | 0 | <MDL | -- | 0 | <MDL | 0 | <MDL | -- |
| **DEHT^d^** | 86 | 2,040 | 78 | 1,890 | 0.53 | 100 | 6,000 | ns | ns | n/a | ns | ns | ns | ns | n/a |

MDL=method detection limit; %>MDL= Percent of chemicals detected above the method detection limit; Med=median; n/a=not applicable; ns=not sampled; <MDL=Concentration was below the MDL.

Missing values indicate that foods were not detected in at least 50% of samples for analyses

^a^Median value represents all food items collected from hamburger chains (hamburgers, French fries, hamburger patties, and chicken nuggets).

^b^Statistical analyses conducted using a Wilcoxon Rank Sum exact test.

^c^Sampled in Phase 1 only (Hamburger Chain A: n=12; Hamburger Chain B: n=14; Tex-Mex Chain A: n=4)

^d^Sampled in Phase 2 only. For hamburger chains, the values represented pertain to hamburgers and fries, as chicken nuggets and hamburger patties were not sampled in Phase 2. Pizza was also not sampled in Phase 2 (Hamburger Chain A: n=7; Hamburger Chain B: n=9; Tex-Mex Chain A: n=6).

**Table S5.** Comparison by phase of median concentrations (μg/kg) of ortho-phthalates and replacement plasticizers in all food types (hamburgers, fries, and chicken burritos) sampled in both phase 1 and 2.

|  | **Phase 1 foods**  **(hamburgers, fries, burritos)**  **n=19** | | **Phase 2 foods**  **(hamburgers, fries, burritos)**  **n=22** | | **p-value^a^** |
| --- | --- | --- | --- | --- | --- |
|  | % > MDL | Median | % > MDL | Median |  |
| **Ortho-phthalates sampled in phases 1 and 2^b^** | | | | | |
| BBzP | 16 | <MDL | 36 | <MDL | -- |
| DnBP | 89 | 2.8 | 100 | 4.8 | 0.005 |
| DEP | 21 | <MDL | 64 | 7.1 | -- |
| DEHP | 74 | 17.0 | 100 | 45.1 | 0.0001 |
| **Replacement Plasticizers sampled in phases 1 and 2^c^** | | | | | |
| DEHA | 21 | <MDL | 100 | 9.4 | -- |
| DINCH | 11 | <MDL | 14 | <MDL | -- |

%>MDL= Percent of chemicals detected above the method detection limit; <MDL=Concentration was below the MDL.

Missing values indicate that foods were not detected in at least 50% of samples for analyses

^a^Statistical analysis conducted using a Wilcoxon Rank Sum exact test for chemicals detected in at least 50% of foods.

^b^DiBP and DiNP are not included in this table, as they were only evaluated in phase 1. DMP and DnOP not shown because all values below MDL.

^c^DEHT is not included as it was only sampled in phase
